# The Dynamic Instability of the Aneuploid Genome

**DOI:** 10.3389/fcell.2022.838928

**Published:** 2022-02-21

**Authors:** Lorenza Garribba, Stefano Santaguida

**Affiliations:** ^1^ Department of Experimental Oncology at IEO, European Institute of Oncology IRCCS, Milan, Italy; ^2^ Department of Oncology and Hemato-Oncology, University of Milan, Milan, Italy

**Keywords:** aneuploidy, cancer, genome instability, mitotic errors, chromosomal instability

## Abstract

Proper partitioning of replicated sister chromatids at each mitosis is crucial for maintaining cell homeostasis. Errors in this process lead to aneuploidy, a condition in which daughter cells harbor genome imbalances. Importantly, aneuploid cells often experience DNA damage, which in turn could drive genome instability. This might be the product of DNA damage accumulation in micronuclei and/or a consequence of aneuploidy-induced replication stress in S-phase. Although high levels of genome instability are associated with cell cycle arrest, they can also confer a proliferative advantage in some circumstances and fuel tumor growth. Here, we review the main consequences of chromosome segregation errors on genome stability, with a special focus on the bidirectional relationship between aneuploidy and DNA damage. Also, we discuss recent findings showing how increased genome instability can provide a proliferation improvement under specific conditions, including chemotherapeutic treatments.

## Introduction

Cell division is the most hazardous stage of the cell cycle, in which a mother cell must accomplish the delicate task to generate two identical daughter cells. This is achieved during mitosis, when chromosomes are segregated between the daughter cells after being duplicated in S phase. The mechanism underlying chromosome segregation is based on the attachment of sister chromatids to the mitotic spindle. Central to this is the proper assembly of a specialized pool of proteins, collectively known as the kinetochore, on centromeric regions of each sister chromatid and subsequent binding to microtubules. Kinetochores built on sister chromatids should bind to microtubules emanating from opposite poles to generate amphitelic attachments and to be faithfully segregated into the two daughter cells (Santaguida and Musacchio, 2009). The fidelity of chromosome segregation is ensured by the spindle assembly checkpoint (SAC), which serves to delay the metaphase-anaphase transition until all chromosomes are properly attached to the spindle. When all faulty attachments have been converted into amphitelic and the unattached kinetochores have been properly bound to the spindle, the SAC is silenced and cells can progress into the cell cycle (London and Biggins, 2014). Importantly, erroneous kinetochore-microtubule attachments lead to chromosome segregation errors and thus to the generation of aneuploid cells, *i.e.*, cells with an abnormal number of chromosomes. Aneuploidy represents a major cause of spontaneous abortions and mental retardation, and is strongly associated with cancer (Santaguida and Amon, 2015; Ben-David and Amon, 2020).

There are several origins of unfaithful chromosome segregation, which can be classified in “pre-mitotic” and “mitotic” (Burrell et al., 2013). The former includes abnormal DNA structures generated by faulty DNA repair and replication that had occurred before mitosis. The latter comprises a variety of mitotic defects, including incorrect kinetochore-microtubule attachments (mentioned above) (Levine and Holland, 2018), aberrant SAC function (Levine and Holland, 2018), altered microtubule dynamics (Bakhoum et al., 2009) (*e.g.,* increased stability of the attachments), mitotic spindle aberrations (*e.g.,* multipolar spindle) (Basto et al., 2008; Maiato and Logarinho, 2014) and cohesion defects (Barber et al., 2008). Although the mechanisms leading to chromosome mis-segregation can vary, its outcome is the generation of a progeny with an unbalanced karyotype, which, in a classical view, harbors segmental aneuploidies in case of pre-mitotic defects and whole-chromosome aneuploidies in case of mitotic defects. However, what has become evident over the last decade is that segregation errors originating from defects of the mitotic machinery not only can lead to gain or loss of entire chromosomes but also to structural chromosomal aberrations (Janssen et al., 2011; Levine and Holland, 2018). This can be explained by the fact that such events often lead to DNA damage (Levine and Holland, 2018).

In this review, we will first discuss how chromosome segregation errors can trigger genomic instability, then will focus on the aneuploid status and its association with replication stress and the subsequent genomic instability. Lastly, we will elaborate on the consequences of genomic instability on aneuploid cell proliferation, including the implications for aneuploid cancer cell physiology.

## Chromosome Segregation Errors Lead to Genomic Instability

Two common by-products of cell division errors are lagging chromosomes in anaphase and generation of micronuclei in the following G1, which both can be associated with DNA damage. In fact, when chromosomes lag behind the main DNA masses for a long time and fail to clear the spindle midzone prior to completion of cytokinesis, they become trapped in the cleavage furrow and could be broken by physical forces (Janssen et al., 2011). Since the trapped genetic material stains positive for γH2AX and MDC1 and cells treated with cytokinesis inhibitors such as blebbistatin display less γH2AX and 53BP1 foci, it can be concluded that DNA damage occurs during cytokinesis. Daughter cells that had inherited broken chromosomes activate a DNA damage response that is typical of cells dealing with double-stranded DNA breaks (DSBs), as shown by the activation of ATM/Chk2 and p53 ^11^. Since inhibition of non-homologous end joining (NHEJ) prevents the resolution of 53BP1 foci, it can be argued that DNA damage induced by chromosome segregation errors is at least partially repaired by NHEJ. This explains why some of the daughter cells harbor unbalanced chromosomal translocations (Janssen et al., 2011).

Alternatively, work from the de Lange and Pellman groups has shown that lagging chromosomes (including the dicentric ones, derived from telomere fusion events and suspected to trigger genomic instability in cancer cells (Artandi and DePinho, 2010)) might not get broken during mitosis, but, instead, form long chromatin bridges between the daughter cells (Martin and Santaguida, 2020). During the first interphase, they can become accessible to cytoplasmatic endonucleases such as TREX1 and get cleaved, exposing ssDNA and facilitating their fragmentation (Maciejowski et al., 2015). Alternatively, they can get stretched by the actomyosin-dependent mechanical force, which also promotes local chromosome fragmentation (Umbreit et al., 2020). In both cases, due to defective replication of the broken ends, the outcome will be the accumulation of additional DNA damage. This results in complex chromosome rearrangements that are compatible with chromothripsis (Maciejowski et al., 2015; Umbreit et al., 2020), a phenomenon in which one or a few chromosomes in a cancer cell harbor several clustered rearrangements (Forment et al., 2012).

Beside lagging chromosomes, micronuclei are also associated with DNA damage. Micronuclei originate from genetic material that had been erroneously segregated and become separated from the daughter cell chromatin masses forming a separate compartment. They can contain whole chromosomes or chromosomal fragments, depending on the nature of the missegregation event (Thompson and Compton, 2011). Increasing evidence indicates that micronuclei are dysfunctional structures, as processes such as DNA replication, transcription, DNA damage repair and nuclear-protein localization exhibit functional defects (Hoffelder et al., 2004; Terradas et al., 2009; Xu et al., 2011; Crasta et al., 2012; Terradas et al., 2012). After mitosis, the genetic material contained in the MN can be re-incorporated into the primary nucleus of the daughter cells at a significant high frequency (Crasta et al., 2012; Soto et al., 2018). This might precipitate cells in a state in which mutations arisen from faulty micronuclear DNA metabolism can be potentially transferred from the MN to the genome.

It is noteworthy that micronuclei are not simple by-products of missegregation events, but could play an active role in triggering and fueling genomic instability (Terradas et al., 2016). Indeed, they often accumulate high levels of DNA damage, which is due to several reasons. First, abnormal replication in micronuclei can directly lead to DNA breaks, as shown by the accumulation of γH2AX foci in the G2 phase (Crasta et al., 2012). Second, if a cell harboring a MN enters mitosis with micronuclear DNA still undergoing DNA replication, chromosomes will compact prematurely and chromosome pulverization will occur (Crasta et al., 2012). Third, the ruptured membrane that is commonly present in micronuclei (Géraud et al., 1989) leads to increased torsional stress and favors chromosome fragmentation (Liu et al., 2018; Vietri et al., 2020). The combined effect of the factors listed above is that DNA in micronuclei can generate a wide spectrum of chromosome rearrangements. By using an elegant approach based on live-cell imaging and single-cell genome sequencing, the Pellman group has shown that some of the events occurring in micronuclei recapitulate chromothripsis (Hatch and Hetzer, 2015; Zhang et al., 2015). This has been recently further characterized by Ly and co-authors, who have been able to dissect the exact categories of genomic rearrangements derived from a single chromosome missegregation event (Ly et al., 2019). Importantly, the fact that the micronuclear membrane is often ruptured leads to spillage of micronuclear DNA into the cytosol (MacKenzie et al., 2017). Thus, the DNA becomes accessible to the cytosolic nucleic acid sensor cGAS, which gets activated and generates the cyclic dinucleotide cyclic GMP-AMP (cGAMP). In turn, cGAMP triggers a type I interferon response *via* STING (stimulator of interferon genes), activating an immune surveillance mechanism. This establishes a direct link between the micronuclei -and therefore genomic instability- and innate immune responses (Harding et al., 2017; MacKenzie et al., 2017). It was already known that STING activation can lead to NF-kB pathway activation (Abe and Barber, 2014), but recently it has been observed that the activation of STING and non-canonical NF-kB pathway can mediate metastasis in a tumor cell-autonomous fashion (Bakhoum et al., 2018). In conclusion, micronuclei can not only exacerbate genomic instability of the cell from which they were generated from, but also favor tumor evolution, and should therefore be considered as highly dangerous structures.

## Aneuploidy Is Associated With Increasing Genome Instability

As previously mentioned, chromosome segregation errors are associated with genome instability and lead to the generation of aneuploid cells. Increasing evidence both in yeast and in higher eukaryotes indicates that the aneuploid status is characterized by additional genome instability. Work from the Amon lab has revealed that missegregation of a single chromosome is sufficient to induce genome instability in yeast (Torres et al., 2007; Sheltzer et al., 2011). In fact, the analysis of 13 aneuploid budding yeast strains has shown that gain of single chromosomes leads to chromosome loss, defective mitotic recombination and DNA damage repair (Sheltzer et al., 2011). As a result, aneuploid strains often enter mitosis in the presence of unrepaired DNA, which can trigger chromosomal translocations (Blank et al., 2015). Similar observations were also made by Zhu and co-authors, who observed that aneuploid yeast strains generated by sporulation of triploid or pentaploid yeast also exhibit chromosomal instability (Zhu et al., 2012).

In line with the data obtained in yeast, work in Chinese hamster embryo and human cells indicates that aneuploidy is associated with genome instability also in higher eukaryotes (Li et al., 1997; Nawata et al., 2011; Nicholson et al., 2015). Indeed, HE35 cells with an extra copy of chromosome 8 display an increase in structural chromosomal aberrations (Nawata et al., 2011). Also, a systematic comparison between trisomic and diploid human cells (both untransformed amniotic fibroblasts and colorectal cancer cells DLD1) has uncovered that aneuploidy is associated with increased frequency of anaphase lagging chromosomes and cytokinesis failure (Nicholson et al., 2015).

Altogether, this shows that chromosome missegregation events promote additional genome instability. This is likely to be due to the major impact of karyotype abnormalities on cellular transcriptome and proteome (Gao et al., 2007; Stingele et al., 2012; Donnelly et al., 2014; Dürrbaum et al., 2014; Santaguida et al., 2015). More specifically, imbalances in the levels of factors critical for fundamental processes such as DNA replication, DNA repair and mitosis are at the basis of the genome instability associated with aneuploidy (Holland and Cleveland, 2012; Chunduri and Storchová, 2019). For example, regarding DNA replication, it has been demonstrated by the Storchova lab that cells harboring extra chromosomes exhibit reduced levels of MCM2-7 proteins, which are essential for the process of DNA synthesis (Passerini et al., 2016). In line with this, analysis of DNA replication dynamics in aneuploid cells has revealed that they experience replication stress in S phase. In fact, aneuploid cells display reduced DNA replication fork rate, increased fork stalling and prolonged S phase duration (Santaguida et al., 2017). All this is indicative of replication stress, which is further confirmed by a high sensitivity of aneuploid cells to replication stress inducing agents such as aphidicolin (Passerini et al., 2016). Importantly, exacerbation of this replication stress can represent a successful strategy to specifically hit cancer cells, which are very often aneuploid. For example, induction of replication fork asymmetry *via* exposure to a PARG inhibitor can selectively kill a subset of ovarian cancer cells (Pillay et al., 2019).

It is well established that faulty DNA replication can trigger genome instability not only by generating DNA damage in S phase but also by challenging the fidelity of chromosome segregation in mitosis. Interestingly, the mechanisms by which this occurs also include stabilization of mitotic spindle microtubules, which favors premature centriole disengagement and generates transient multipolar spindle (Böhly et al., 2019; Wilhelm et al., 2019). In line with the impact of replication stress on genome stability, Burrell, McClelland and co-authors have found that replication stress is what triggers structural and numerical chromosomal instability (CIN) in most colorectal cancers, thus challenging the classical view that only mitotic defects can lead to numerical aneuploidy (Burrell et al., 2013). In conclusion, aberrant DNA replication appears to be the main factor contributing to a further increase in aneuploid cell genome instability. It is likely that both genomic imbalances caused by the aneuploid status and previously-mentioned replication problems in micronuclei contribute to the replication defects displayed by aneuploid cells. Collectively, they lead to the accumulation of additional DNA damage (Santaguida et al., 2017), thus triggering further genome instability in aneuploid cells.

## Consequences of Genomic Instability on Aneuploid Cell Fitness

As previously mentioned, karyotype imbalances have important consequences on cellular transcriptome and proteome. More in details, there is a direct dosage effect on the expression of genes present on aneuploid chromosomes, *i.e.,* RNA expression from the gained chromosomes is proportional to chromosome copy number both in yeast and in higher eukaryotes (Torres et al., 2007; Williams et al., 2008; Pavelka et al., 2010) -with some exceptions (Rancati et al., 2008; Letourneau et al., 2014). Similarly, changes in protein abundance tend to roughly scale with changes in DNA copy number in yeast (Pavelka et al., 2010; Sheltzer et al., 2011; MacKenzie et al., 2017). However, this is not always the case: when translation rate was assessed in 12 disomic yeast strains, it was found that around 20% of proteins was synthesized at a lower rate than predicted based on copy number changes. These proteins were mostly components of multi-subunit complexes (Dephoure et al., 2014). Similarly, in human aneuploid cells, the abundance of this type of proteins and protein kinases was found to be reduced toward diploid levels (Stingele et al., 2012). Collectively, this could indicate that either some genes on aneuploid chromosomes are not translated efficiently or their products are not stable. The Amon lab has proposed that this “dosage compensation” occurs *via* the activation of proteolytic pathways, so that cells can compensate for abnormal protein stoichiometry (Torres et al., 2008; Sheltzer et al., 2011; Pfau and Amon, 2012). And this was also confirmed for multimolecular complexes (McShane et al., 2016).

Given the deep impact of karyotype abnormalities on cell physiology, it is not surprising that aneuploidy is detrimental for cellular fitness (Santaguida and Amon, 2015; Zhu et al., 2018). This is evident not only in budding and fission yeast, where aneuploid strains proliferate at a slower rate than controls (Torres et al., 2007; Holland and Cleveland, 2012), but also in mouse, where trisomy of chromosome 1, 13, 16 or 19 is associated with proliferation defects in MEFs (Williams et al., 2008). In humans, all monosomies are lethal and only three autosomal trisomies are viable: chromosome 13, 18 and 21, which are the poorest gene-containing chromosomes (Holland and Cleveland, 2012). Only individuals with trisomy 21 survive until adulthood and their cells are well known to proliferate at a slower rate than age-matched diploid cells (McCoy et al., 1983). Instead, aneuploidies of sex chromosomes are better tolerated than aneuploidies of autosomes, probably because the Y chromosome encodes for a few genes only and only one X chromosome is active in adult cells regardless of how many copies are present. It is important to highlight that, beside gain of single chromosomes, sometimes cells can acquire more complex karyotype abnormalities. When aneuploidy becomes severe, cells do not simply slowdown in the cell cycle, but activate p53 and undergo consequent cell cycle arrest (Santaguida et al., 2017).

Intriguingly, although aneuploidy is usually deleterious for cell physiology, some human tissues such as brain and liver naturally contain aneuploid cells (Pack et al., 2005; Duncan et al., 2012; Santaguida and Amon, 2015). Although the biological significance of aneuploidy in these contexts has not been elucidated yet, it can be speculated that aneuploidy could allow liver cells, for example, to adapt to nutritional and noxious stresses (Holland and Cleveland, 2012). This seems to be particularly relevant to cancer cells, which are found to be very often aneuploid (Vasudevan et al., 2021). In fact, recent work has shown that abnormal chromosome number in the context of cancer can promote resistance to chemotherapy (Salgueiro et al., 2020; Ippolito et al., 2021; Lukow et al., 2021), in line with the observation that highly aneuploid tumors correlate with poorer patient outcomes (Davoli et al., 2017; Vasudevan et al., 2020). In details, chromosome missegregation events cause increased karyotypic heterogeneity that can be utilized by cancer cells to find the correct karyotypic landscape and thus survive under selective pressures such as chemotherapy. In conclusion, under stress-free conditions, aneuploidy causes decreased proliferation, while under suboptimal conditions aneuploid cells might grow better. This provides an explanation for the apparent paradox of cancer cells, which are very often aneuploid and at the same time are characterized by increased proliferation.

One final aspect that deserves to be discussed is the role that aneuploidy-induced CIN plays in tumorigenesis (Funk et al., 2016; van Jaarsveld and Kops, 2016). In the previous section, we mentioned that aneuploidy is frequently associated with increased genome instability. An increased DNA mutation rate can favor the onset of genetic alterations that drive cellular growth and transformation (Holland and Cleveland, 2012). Recent studies in mouse have demonstrated that CIN can favor the expansion of cells with clonal chromosomal abnormalities, which act as tumor-initiating cells (Levine et al., 2017; Shoshani et al., 2021; Trakala et al., 2021). This is the case of chromosome 15, on which the oncogene Myc is located, and was found to be gained with high prevalence in a mouse model of T-cell lymphoma (Shoshani et al., 2021; Trakala et al., 2021). Interestingly, expression of human MYC from chromosome 6 leads to karyotype changes, namely gain of chromosome 6. Under this condition, chromosome 15 is still frequently gained, unless the Rad21 gene is deleted from it (Trakala et al., 2021). This shows that clonal selection is guided by chromosomal location and identity of specific genes.

## Concluding Remarks

**FIGURE 1:**
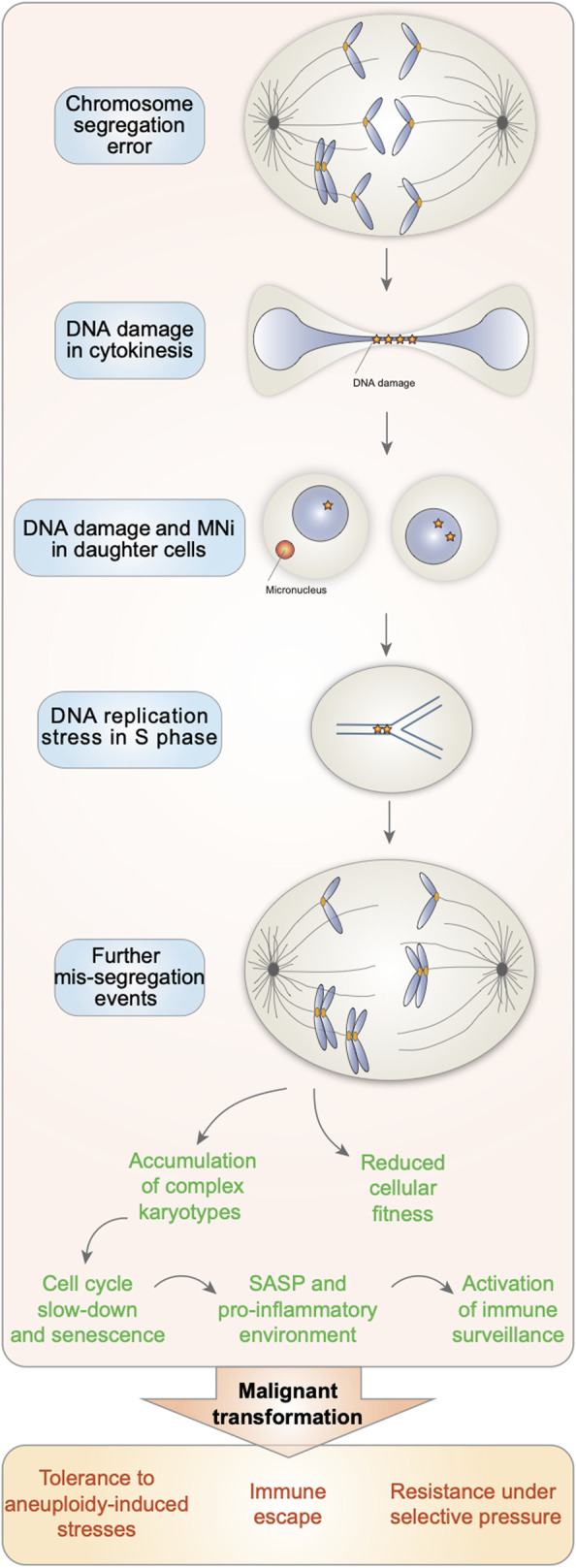
Aneuploid cells are characterized by increasing genome instability. Chromosome segregation errors lead to the generation of aneuploid cells with DNA damage. When attempting to duplicate their genome in S phase, aneuploid cells experience DNA replication stress. Altogether, this triggers further missegregation events in the subsequent cell cycles and thus the accumulation of cells with complex karyotypes, known for displaying reduced cellular fitness, entering senescence and displaying a senescence associated secretory phenotype (SASP). However, in the context of cancer, the increasing genome instability associated with aneuploidy can confer a proliferative advantage. This would allow them to survive and provide a strong advantage in the presence of selective pressures, such as during chemotherapy.

In this review, we have discussed how chromosome segregation errors induce DNA damage and how this exacerbates genome instability in the resulting aneuploid cells, a condition that has recently been shown to confer proliferative advantages under specific conditions (Ippolito et al., 2021; Lukow et al., 2021; Salgueiro et al., 2020) (Figure 1). It has to be noted that the vast majority of studies conducted so far have employed an heterogenous population of aneuploid cells, comprising both chromosome gains and losses as well as cycling and arrested cells. In the future, it will be important to explore more in details the contributions of specific trisomies *vs.* monosomies, an effort that has been recently pioneered by the Medema and Storchova labs (Chunduri et al., 2021; Hintzen et al., 2021). Further, it remains unclear what are the exact mechanisms by which the aneuploid status is associated with increasing genome instability. Although aneuploid cells usually proliferate at a slower rate than euploid counterparts and eventually cease to divide, some cells with karyotype imbalances can keep cycling. It would be particularly interesting to assess the levels of genome instability in this cycling sub-population of cells and investigate by which mechanisms they can cope with it and remain capable to proliferate. Current available methodologies do not allow for the recovery of a population of cycling aneuploid cells suitable for further analyses (since those methodologies employ spindle poisons to separate the cycling counterparts (Santaguida et al., 2017; Wang et al., 2018; Wang et al., 2021)). Therefore, in the future it would be very important to develop new strategies to separate arrested and cycling aneuploid cells. Since DNA replication seems to be a major contributing factor to aneuploidy-induced genome instability, we speculate that the efficiency of DNA replication and DNA repair processes would be different between arrested and cycling aneuploid cells, and that this would be the key factor in determining the fate of cells harboring chromosome imbalances. Gaining this knowledge will uncover novel dependencies of aneuploid cells with the exciting possibility to exploit them to selectively eradicate aneuploid tumors.
